# B7 Family Molecule VSIG4 Regulates Intestinal Anti-*Enterohemorrhagic Escherichia coli* Immunity by Altering Gut Flora Diversity

**DOI:** 10.3390/microorganisms9081769

**Published:** 2021-08-19

**Authors:** Zhili He, Jiajia Li, Saisai Gong, Li Xing, Yakun Sun, Jianxin Wang, Tao Li, Nianzhi Ning, Liangyan Zhang, Wenjing Yu, Deyan Luo, Hui Wang

**Affiliations:** State Key Laboratory of Pathogens and Biosecurity, Beijing Institute of Microbiology and Epidemiology, Beijing 100071, China; lili892017173@163.com (Z.H.); 13024096923@163.com (J.L.); gby1738131995@163.com (S.G.); spring@aliyun.com (L.X.); syk_dyx@hotmail.com (Y.S.); jianxinwang1994@163.com (J.W.); litaobmi@126.com (T.L.); ningnianzhi@163.com (N.N.); polini@live.cn (L.Z.); cpu_ywj@163.com (W.Y.)

**Keywords:** VSIG4, B7, EHEC, intestinal microorganism, intestinal immunity

## Abstract

As an essential member of the B7 family, V-set and immunoglobulin domain-containing 4 (VSIG4) is expressed explicitly in tissue-resident macrophages (TRMs) and plays an essential role in maintaining the homeostasis of the environmental immune system. Here, we demonstrate that gene-targeted VSIG4-deficient mice infected with *Enterohemorrhagic Escherichia coli* (EHEC) display reduced bacterial burden. To reveal the role of VSIG4 in the fight against EHEC infection, we collected mice feces and used high-throughput 16S rRNA gene amplicons to detect changes in the flora. A total of 657330 sequences were sequenced on the PacBio platform, with an average length of 1498 bp. We found that VSIG4 deficiency could alter the gut microbiota by increasing diversity and shifting community composition. In particular, *G_Akkermansia* and *G_Oscillo spiraceae* increased significantly. These findings expand upon a prior observation that VSIG4 deficiency reduced EHEC colonization by changing the gut microbiota diversity and shifting community composition.

## 1. Introduction

V-set and immunoglobulin domain-containing 4 (VSIG4) is a multifunctional cell surface protein with effective immunosuppression and anti-inflammatory effects [1]. The expression of VSIG4 is restricted to the tissue-resident macrophages (TRMs) and is a marker of this subset of Mφ. The phenotypic characteristics of these macrophages are CD11b^+^ F4/80^hi^ VSIG4^+^. VSIG4^+^ Mφ can be detected in the large intestine, liver, lung, heart, and peritoneal cavity [2,3,4,5]. As a member of the B7 superfamily, VSIG4 can be used as a co-suppressor to inhibit the proliferation of T cells and the production of IL-2 cytokines [6,7]. Recent research indicates that VSIG4 exhibits the dual functions of inhibiting early T cell activation and promoting the differentiation of Treg cells [4]. Given its immunosuppressive function, some scientists have proved that VSIG4 can promote the occurrence and progression of cancer [8,9,10]. In addition, VSIG4 is also a complement receptor. It can bind to C3b, iC3b, and C3c and participate in the phagocytosis of microorganisms, which plays a central role in the rapid clearance of circulating pathogens [3,11,12,13]. Tanaka et al. found VSIG4^+^ Mφ in the lamina propria of colorectal mucosa in mice and humans, and a large proportion of the Mφ-dependent C3b phagocytosis of the large intestine was found to be involved in VSIG4 [5]. The importance of VSIG4 in eliminating foreign bodies in the large intestine was emphasized. We speculate that the disappearance of this phagocytosis may provide a suitable colonization environment for a variety of microorganisms.

*Enterohemorrhagic Escherichia coli* (EHEC) is an important food-borne pathogen that mainly colonizes the host colon and causes hemorrhagic diarrhea. Severely ill patients will develop hemolytic uremia and renal failure [14]. EHEC is extremely viable in the environment and can persist for long periods of time. It is resistant to strong acids and can cause disease at low doses [15]. After entering the human body, EHEC adheres to the colon wall to reproduce and is excreted through feces. Contaminated water and vegetables have caused several outbreaks around the world. The most influential outbreak occurred in Germany in 2011. There were reported cases in eight countries, including countries in Europe and North America, among which 53 cases were fatal. At present, there is no effective treatment for EHEC infection, and EHEC is mainly controlled through prevention. EHEC faces complex and shifting challenges when it invades the intestine, and the immune system of the intestine is an important checkpoint. The gastrointestinal tract contains a large amount of secondary lymphoid tissue, accounting for about 70% of the entire immune system. This means that the intestinal mucosa is the largest lymphoid organ in the body [16,17]. In the gastrointestinal tract, lymphocytes coexist harmoniously with about 3.8 × 10^13^ symbiotic bacteria [18]. The intestinal mucosa contains a large number of macrophages, and scientists have found that the VSIG4 molecule is universally expressed in the gastrointestinal tract of mice [3,5,6].

Therefore, this article discusses whether VSIG4 plays a role in clearance and resistance during EHEC infection. Surprisingly, as a complement receptor, the lack of VSIG4 does not weaken the host’s clearance ability but enhances the host’s resistance to EHEC. We further examined the changes in the intestinal microbiome before and after VSIG4 deletion and found that VSIG4 changed the intestinal anti-EHEC immunity by regulating the intestinal flora. Thus, VSIG4 inhibitor can be used as a new treatment for EHEC infection.

## 2. Materials and Methods

### 2.1. Animals and Treatments

*Vsig4* knockout mice with C57BL/6J as the background were produced by the Laboratory Animal Center at the Academy of Military Medical Sciences, Beijing. The CRISPR/Cas9 gene editing technique was used to target and knockout 624 bp in exon 2 (the amplification primers used for verification were F: cacacaatagctagcacaaccagtg and R: aaattgggagatgtacttggtggga). The quantitative real-time PCR (qRT-PCR) primers for *Vsig4* mRNA verification were F: gtgaaatggctggtaagacacg and R: cagcaaagggagatgatgaaga. The mice were housed in transparent plastic cages with no more than five mice in each cage. The mice were supplied with deionized water and SPF grade feed. The environment was strictly controlled, with a 12:12-h light-dark cycle at constant room temperature (25 °C).

The *Vsig4*^+/−^ gene (Het, Heterozygote) mice and *Vsig4*^−^^/−^ gene (KO, knockout) mice were randomly selected at six–eight weeks. The mice were fed for another week to adapt to the grouping environment. Three to six pieces of fresh feces were collected from each mouse and placed into clean Eppendorf tubes. The tubes were marked and then frozen in liquid nitrogen and stored at −80 °C. This study was carried out according to the recommendations set out in the Guide for the Beijing Institute of Microbiology and Epidemiology Animal Care and Use Committee (IACUC number. 2020-011).

### 2.2. DNA Extraction and Sequencing

Genomic DNA in feces was extracted using the cetyltrimethylammonium bromide (CTAB) method [19], and then, the purity and concentration of DNA were detected by agarose gel electrophoresis. The samples were diluted to 1 ng/μL with sterile water in centrifuge tubes. Genomic DNA was used as a template to sequence the 16S full-field. The specific primers with Barcode were as follows: 16S-F: AGAGTTTGATCCTGGCTCAG and 16S-R: GNTACCTTGTTACGACTT. We used Phusion^®^ High-Fidelity PCR Master Mix with GC Buffer and high-performance high-fidelity enzymes (New England Biolabs, Beijing, China) for PCR. The sequencing linker was connected to both ends of the amplified DNA fragment by DNA ligase, and the DNA fragment was purified and selected by AMpure PB magnetic beads to construct the SMRT Bell library. After the purified fragments were dissolved by elution buffer, the fragments of specific size were screened by BluePipin fragments, and the DNA fragments were purified by AMpure PB magnetic beads. The constructed library was quantified by Qubit concentration, and the size of the inserted fragment was detected by Agilent 2100 and then sequenced using the PacBio sequel I platform.

### 2.3. Bioinformatic Data Processing

PacBio offline data were exported to a file in BAM format. *lima* (https://lima.how/) was used to distinguish the data of each sample according to the barcode sequence, and the sequences of all samples were saved in BAM format. CCS (SMRTLinkv7.0) was then used to correct the sequences, and the correction parameters were as follows: CCS = 3, and the minimum accuracy was 0.99. The sequences with lengths less than 1340 bp and greater than 1640 bp were removed and saved with fastq and fasta. SSR filtering was then performed, and the primers were removed using Cutadapt; the sequences with the same consecutive base number > 8 were filtered out as well. The read sequences were compared with the full-length annotation database to detect the chimera sequences (UCHIME Algorithm) (Bioinformatics 27.16 (2011): 2194–2200), and the chimera sequences were removed (Genome research 21.3 (2011): 494–504.) and the final clean reads were obtained. The original sequencing data were uploaded to the NCBI database (https://www.ncbi.nlm.nih.gov/sra/PRJNA734571 (access on 15 June 2021) BioProject ID: PRJNA734571).

The operational taxonomic unit (OTU) clustering steps were as follows: (1) the non-repetitive sequences were extracted from the optimized sequences; (2) the single sequences without repetition were removed; (3) non-repetitive sequences (excluding single sequences) were clustered according to 97% similarity, and chimerism was removed in the clustering process to obtain representative OTU sequences; (4) all optimized sequences were mapped to OTU representative sequences, and sequences with more than 97% similarity to representative sequences were selected. In order to obtain the species classification information corresponding to each OTU, the RDP classifier Bayesian algorithm was used to analyze the 97% similarity OTU representative sequences, and the community species composition of each sample was counted at each classification level.

We used R language tools to create the rank-abundance curve, Venn diagram, community column chart, community heatmap, PCoA, and NMDS diagrams. The Shannon, Simpson, and Chao indexes and PD_whole_tree and UniFrac distance were calculated by Qiime software (Version 1.9.1, https://github.com/biocore/qiime/blob/master/CONTRIBUTING.md). The relationship between sample and species was plotted by Circos-0.67-7 (http://circos.ca/). Linear discriminant analysis (LDA) effect size (LEfSe) software was used for the linear discriminant analysis of samples according to different grouping conditions (LDA) in order to reveal the significant differences in the sample division of the community or species.

### 2.4. Construction of Mouse Fecal Microbiota Transplantation (FMT) Model

The Het and KO mice were randomly selected and fed for another week to adapt to the grouping environment. A group of KO mice (*n* = 5) were given a broad-spectrum, malabsorption antibiotic cocktail (0.5 g/L vancomycin, 1 g/L neomycin sulfate, 1 g/L metronidazole, and 1 g/L ampicillin) in drinking water for one week to deplete the intestinal microflora. The antibiotics were stopped, and the feces of the Het mice were collected and suspended in phosphate buffer at a concentration of 0.125 g/mL. A 200 μL of fecal suspension was used for gavage into each mouse, once a day, for seven days. A group of KO and Het mice were supplied with distilled water and intragastric gavage with PBS as control. At day 0, 7, and 14, feces were collected to make a suspension and cultured on Columbia blood plates to verify the intestinal microbial removal effect and the bacteria colonization effect.

### 2.5. Mice Infection

Six *Vsig4*^+/−^ gene mice and six *Vsig4*^−/−^ gene mice were randomly selected. EHEC strains were cultured overnight in fresh LB medium, and mice were infected intragastrically with a dose of 1 × 10^9^ per mouse to establish an EHEC infection model. Feces were collected and weighed at 12, 24, 36, and 48 h. The feces were dissolved in PBS, and the volume was fixed to 1 mL. After gradient dilution, the fecal suspension was evenly applied to the Sorbitol-MacConkey agar plates and then incubated at 37 °C for 36 h for counting. At 36 h of infection, we collected the serum and detected the level of keratinocyte-derived cytokine (KC) using a mouse KC ELISA kit (EMC104.96, NeoBioscience, Beijing, China). In addition, we take out the colon tissue, then fixed, sliced, and stained. Scoring criteria such as edema, inflammatory infiltration, necrosis, hemorrhage, etc. were set and sent to pathologists for analysis and score.

### 2.6. Absolute Quantitative Analysis

We designed primers (OSC5 F and R) for the known sequence of *Oscillospira* and synthesized *Akkermansia*-specific primers (Mus F and R) [20] (Supplementary Table S1). We used 1 ng/μL fecal genome as templates and used primers and PCR mix (TSE006, Beijing TsingKe Biotech Co., Ltd., Beijing, China) to amplify the target gene (98 °C initial denaturation, 2 min; 98 °C 10 s, 60 °C 10 s, 72 °C 10 s, 30 cycles; final extension, 72 °C, 2 min). The PCR products after nucleic acid gel recovery and purification (EG101-01, Tran^®^, Beijing, China) were used as standards to be tested for concentration. And then according to the formula [(6.02 × 10^23^ copies/mol) × (concentration g/mL)/(MW g/mol) = copies/mL] to calculate the copies of the target gene (Supplementary Table S1). Next, we use the easy dilution solution to dilute the standard by ten times, and obtain a total of nine gradients. Perform qRT-PCR with nine sets of standard dilutions of each of the 2 genes. The reaction system is: Top Green qPCR SuperMix (AQ131-01, Tran^®^, Beijing, China) 10 μL, front and rear primers (10 μmol/L) each 0.5 μL, template 1 μL, and sterilized ultrapure water 8 μL to a total volume of 20 μL. Three repetitions for each sample were performed. Reaction conditions: 94 °C pre-denaturation for 30 s; 94 °C 5 s, 60 °C 30 s, 40 cycles. We then took five samples of fecal genome from Het and KO group, respectively, and performed qRT-PCR with Mus or OSC5 primers. After obtaining the thershold cycle (Ct) of each gene, calculated it according to the linear formula, and then checked the inverse log table to get the copy number of each target gene. The (MW g/mol) for dsDNA = (number of bps) × (660 Daltons/base). The amplification efficiency E% = (10^−1/slope^ − 1) × 100%.

### 2.7. Statistical Analysis

The box chart of Beta diversity group difference analysis was obtained by *t*-test. The α diversity parameters were tested by non-parametric Mann-Whitney U test. The *R* value of ANOSIM analysis was calculated according to the formula, and then the samples were replaced and the R* value was recalculated. The probability that R* is greater than *R* is the *p* value. The Wilcoxon rank-sum test was used to judge whether the utilization of species abundance between groups was statistically significant. Student *t*-test was used to test the difference of serum KC level between the two groups. *p*-value < 0.05 was considered statistically significant.

$$R=\frac{\bar{\gamma b}-\bar{\gamma \omega }}{\frac{1}{4}[n(n-1)]}$$

## 3. Results

### 3.1. The Vsig4^−/−^ Mice Show Stronger Resistance to EHEC Infection

**Figure 1:**
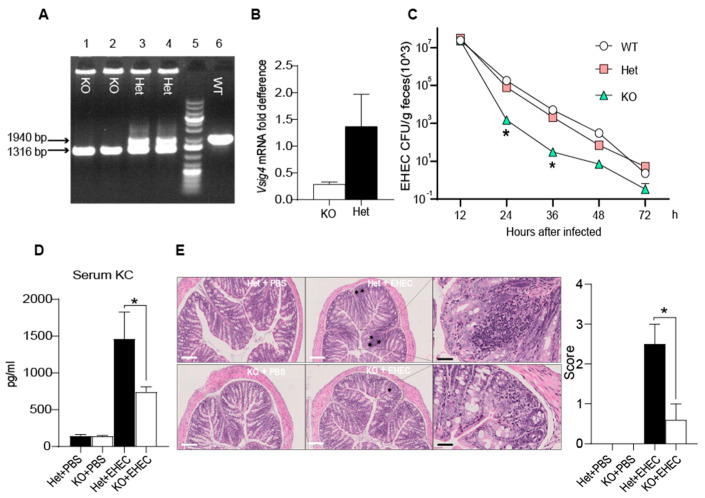
EHEC showed lower colonization in *Vsig4*^−/−^ mice. (**A**) Verification of Vsig4 gene deletion. The tails of WT (*Vsig4*^+/^^+^), Het (*Vsig4^+^*^/−^) and KO (*Vsig4*^−/−^) mice were collected and used as templates for PCR fragment amplification after lysis. The WT (lane 6) has a band size of 1940 bp, while the KO (lane 1, 2) was 1316 bp. And the Het (lane 3, 4) has both bands. (**B**) The total RNA of lung tissue was extracted and used as a template for RT-PCR amplification to verify the mRNA level of Vsig4. Het mice were used as a positive control. (**C**) EHEC infected the mice at a dose of 1 × 10^9^. The EHEC excretion in feces at different time points was detected. (**D**) At 36 h of infection, mice serums were collected and ELISA detected K.C. (IL-8) levels. PBS treated mice served as a control. (**E**) The colons of mice treated at 36 h in each group were sectioned, fixed, and stained. Inflammatory cells (star) were marked (**left**). White bars represent 200 µm, and black bars represent 50 µm. **Right** is the colon injury severity score. The degree of colonic pathological injury ranged from the slightest to the most severe, with a score of 0–10. Three to five fields of vision were selected for each slide *t*-test, * *p* < 0.05, error bars represent SEM. All the above experiments were repeated more than twice. WT: Wild Type; KO: Knockout; Het: Heterozygote; EHEC: *Enterohemorrhagic Escherichia coli*; K.C.: keratinocyte-derived cytokine.

In order to explore whether VSIG4 molecule play a role in the process of EHEC infection, we constructed and identified *Vsig4* knockout mice (Figure 1A,B). Next, we infected mice with EHEC, and the feces of mice were collected at different time points to count the number of EHEC. An unexpected phenomenon occurred. The loss of VSIG4 did not weaken the body’s elimination of pathogenic bacteria, but strengthened the body’s resistance to EHEC colonization. As shown in Figure 1C, the EHEC loads in KO mice gut were lower than that of Het group and WT group, while the difference was significant at 12 h and 36 h. There was no significant difference between the WT and Het groups. Considering Het is a more rigorous control, our subsequent analyses were based on the comparison between Het and KO group. At 36 h, the inflammatory cytokines KC (keratinocyte-derived cytokine, IL-8) in serum were detected, which is the main inflammatory factor response to EHEC infection [21,22]. We found that the KC in infected mice increased significantly, and the level of inflammatory reaction in Het infection group was significantly higher than that in KO infection group (Figure 1D), which was consistent with the colonization of pathogens. In addition, we took out the colons and fixed the section to evaluate the pathological lesions at 36 h. The structure of the intestinal wall, intestinal mucosa glands and gland cells were normal in the two PBS control groups. And there were distinct pathology lesions in Het infection group, such as the intestinal cavity showing infiltration of inflammatory cells in the acinus lamina and local edema. Only a few inflammatory cells were observed in KO infection group (Figure 1E—left). The injury severity scores clearly showed that colons inflammation in KO infection group were much milder (* *p* < 0.05) compared with that in the Het infection group (Figure 1E—right), which indicating that less EHEC colonized colon and caused slighter pathological damage in KO mice.

### 3.2. The Absence of Vsig4 Increases the Species Diversity of Intestinal Microorganisms

Although complement receptors can help the host eliminate pathogenic bacteria, the absence of VSIG4 reduces the colonization of pathogenic bacteria, which aroused our curiosity. Therefore, we collected the feces of KO and Het mice and analyzed the microbiome. We extracted DNA and amplified the full length of 16S after quality testing. After the construction of the SMRT Bell library, a total of 657,330 sequences were sequenced on the PacBio platform, with an average length of 1498 bp. According to 97% similarity, OTU clustering was performed on non-repeated sequences, and then, species annotation was performed on OTU representative sequences to obtain species information and abundance distribution. Next, we analyzed the abundance at different taxonomic levels, the alpha diversity, Venn diagrams, and other analyses of OTUs.

**Figure 2:**
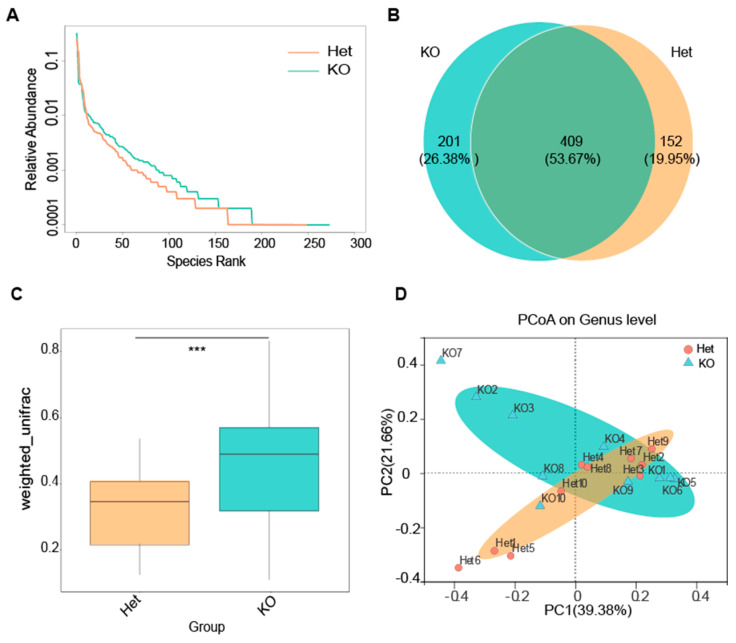
Deletion of the *Vsig4* gene increased intestinal microbial diversity in mice gut. (**A**) KO mice have more abundant microbial species in gut. The abscissa of the curve is the serial number sorted by OTUs abundance, and the ordinate is the relative abundance of the corresponding OTUs. The higher the species richness, the larger the span of the curve on the horizontal axis; the smoother the curve, the more even the species distribution. (**B**) Venn diagram based on OTU. The number in the overlapping part represents the number of OTUs shared by the two groups, and the number without the overlapping part represents the number of unique OTUs in the group. (**C**) Box plot for analysis of differences between groups in Beta diversity, tested by *t*-test, *** *p* < 0.001, error bars represent SD. (**D**) PCoA shows the differences in the community composition of the samples at the genus level. KO, Knockout; Het, Heterozygote; OUT, Optical Transform Unit; PCoA, Principal coordinates analysis.

As shown in Supplementary Figure S1A, with the increase of the number of samples, the number of covered species tended to be flat, indicating that the samples were sufficient for data analysis. Although the sample complexity analysis (alpha diversity analysis) revealed no significant difference between the two groups (Supplementary Figure S1B), the KO group curve in the rank-abundance curve (Figure 2A) had a larger horizontal span than the Het group and the decrease was more gradual, suggesting that the intestinal microbial species diversity and distribution in *Vsig4*^−/−^ mice were more abundant and uniform. In addition, the Venn diagram (Figure 2B) drawn based on OTUs revealed that the two groups were mainly composed of shared OTUs, while the KO group had more unique OTUs.

The beta diversity considers the difference in bacterial community composition for different groups. We choose the weighted UniFrac distance to measure the dissimilarity coefficient of the sample, and there was a significant difference (*p* < 0.001) between the two groups (Figure 2C). A scatter plot based on our principal coordinate analysis (PCoA) (Figure 2D) and 2-D non-metric multidimensional scaling (NMDS) (Supplementary Figure S2) revealed that the feces microbiota in KO mice were different from those identified in the Het mice.

The above results suggest that the deletion of *Vsig4* gene can significantly alter the intestinal flora and increase the diversity of intestinal flora in mice.

### 3.3. Differentially Abundant Phyla and Genera in Gene Deleted Mice and Heterozygous Mice

**Figure 3:**
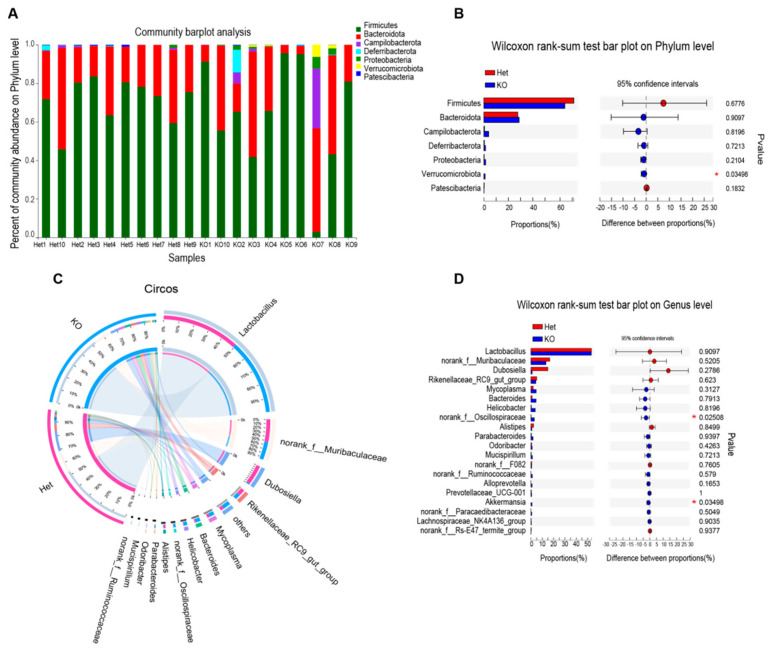
Differentially abundant Phyla and Genera in KO and Het mice. (**A**) Columnar cumulative graph of relative abundance of Phylum species. Others represent the sum of the relative abundances of all the other phyla except for these five phyla in the figure; (**B**) Wilcoxon rank-sum test bar plot on Phylum level. *Verrucomicrobia* in the KO group has a significant increase; (**C**) the relationship between samples and the species at the Genus level. The small semicircle (left half-circle) indicates the composition of the species in the sample. The color of the outer ribbon represents which group it comes from, the color of the inner ribbon represents the species, and the length represents the relative abundance in the corresponding sample; the large semicircle (the right half circle) represents the distribution of species in different samples at the taxonomic level. The outer ribbon represents the species. The inner ribbon color represents different groups. The length represents the proportion of distribution in a species. (**D**) Wilcoxon rank-sum test bar plot on the genus level. The G_*Akkermansia* and G_*Oscillospiraceae* increased significantly in the KO group. * *p* < 0.05. KO: Knockout; Het: Heterozygote.

Based on the results of OTU species annotation, we generated a relative abundance histogram (Figure 3A) in order to determine the dominant phyla and their proportions. Among the top five phyla, the abundance of *Firmicutes*, *Bacteroidetes*, and *Campilobacterota* accounted for 95% of the bacteria. In the heterozygous mice, bacteria were mainly composed of *Firmicutes* (71.27%) and *Bacteroidetes* (27.65%). In the KO group, bacteria from *Firmicutes* (63.87%), *Bacteroidetes* (28.8%), and *Campilobacterota* (3.78%) dominated. The KO group had another phylum, *Verrucomicrobiota* (0.93%), which had an abundance of 0% in the Het group. A heat map of phylum distribution for each sample is shown in Supplementary Figure S3A. We then conducted a statistical test on the differences in bacterial abundance between the two groups (Figure 3B). The distributions of all the phyla were different, and the difference in *Verrucomicrobiota* was significant (*p* < 0.05).

According to genus-level profiling, OTU could be assigned to 34 individual genera. The top five predominant genera in the Het group were *Lactobacillus* (53.35%), *Muribaculaceae* (16.55%), *Dubosiella* (14.85%), *Rikenellaceae* (5.14%), and *Mycoplasma* (1.993%). However, in the KO group, the rankings and proportions of the top five dominant bacteria changed and were *Lactobacillus* (53.25%), *Muribaculaceae* (13.22%), *Rikenellaceae* (4.327%), *Mycoplasma* (4.602%), and *Bacteroides* (4.474%) (Figure 3C). Wilcoxon rank sum tests (Figure 3D) showed that the abundances of *Oscillospiraceae* and *Akkermansia* in the KO group were significantly higher than those in the control group. A heat map of different genera abundances for each sample is shown in Supplementary Figure S3B.

### 3.4. The Significant Different Species between Gene Deletion Mice and Heterozygous Mice

**Figure 4:**
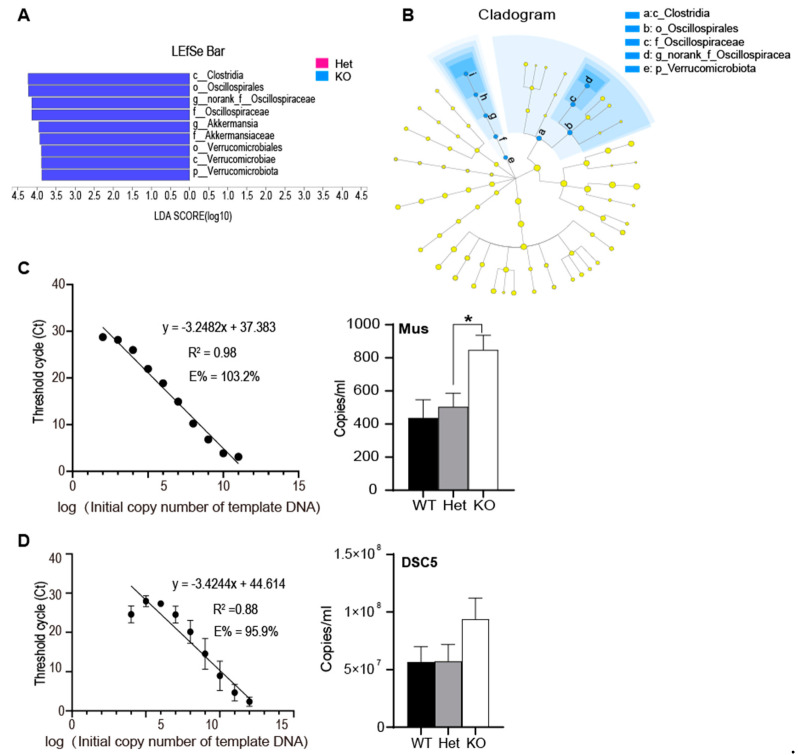
LEfSe analysis of significant differences in species between groups. (**A**) LDA value distribution histogram, the length of the histogram represents the impact of different species (LDA Score); (**B**) LEfSe multi-level species tree diagram: species with significant differences between groups. Nodes with blue indicate microbial groups that are significantly enriched in the corresponding group and have a significant impact on the differences between groups (LDA > 3); light yellow nodes indicate that there is no significant difference in different groups. From the inside to the outside, it is from the phylum to the genus level. LefSe, Linear discriminant analysis effect size. (**C**,**D**) Standard curve (**left**) and copy values of gene fragments in different groups (**right**). *t*-test, * *p* < 0.05, error bars represent SEM. WT, Wild Type; KO, Knockout; Het, Heterozygote.

LDA Effect Size (LEfSe) is a kind of software used to reveal the characteristics of genomes. First, non-parametric factorial Kruskal–Wallis (KW) sum-rank test was used to detect significant abundance differences. And then linear discriminant analysis (LDA) was used to estimate the effect of abundance of each species on the difference (Figure 4A). The LDA was set to >3, and the significant difference with abundance was marked in color (Figure 4B). And this was consistent with the results of Figure 3.

The nominal *p*-value were significant for *Akkermansia* and *Oscillospira*. Although they did not survive multiple testing corrections (Supplementary Difference test statistics table), we validated the abundance change using the absolute quantitative qPCR assays. We used the fecal genome as templates to amplify standards containing specific fragments of *Oscillospira* or *Akkermansia*, respectively. The standard curves shown in Figure 4C,D were drawn according to the Ct (Supplementary Figure S4) and log (initial copy number in template DNA) values of different dilution standards. The R^2^ of the two groups were 0.98 and 0.88, respectively, which have good linear correlation and repeatability, and meet the requirements of the standard curve. Different groups of genomes were used as templates to perform qRT-PCR to obtain the Ct value of specific fragments. After formula conversion, the target gene copies were obtained (Figure 4C,D—right). It shown that the copies of *Akkermansia* were lower in groups, while the number of copies of *Oscillospira* were high. This should be related to their abundance in the gut microbial community. Both *Akkermansia* and *Oscillospira* have an upward trend in the KO mice, while *Akkermansia* increased significantly (* *p* < 0.05).

Due to the anti-inflammatory and immunostimulatory properties, and the ability to improve intestinal barrier function, *Akkermansia* has been considered as a marker of healthy intestinal tract in recent years [23]. It is a mucocolonization bacterium that can use mucus as the only source of carbon and nitrogen [24]. The activity of *Akkermansia* on the mucous membrane can help maintain the morphology of the mucus layer. Therefore, human *Akkermansia* is very important human intestinal tract. At the same time, *Akkermansia. muciniphila* can produce acetate and propionate, which are close to the epithelial cells (50mm), so it is easy for the host to obtain. These products can also stimulate the interaction of microbiota and host response [25]. Oligosaccharides and acetate stimulate the growth and metabolic activity of bacteria, which may provide colonization resistance for pathogenic bacteria to intestinal cells.

Previous studies have shown that there is a decrease in the number of *Oscillospiraceae* in inflammatory diseases [26], and it may contribute to the formation of secondary bile acids and prevent *Clostridium difficile* infection [27]. Although it is very difficult to isolate and culture the *Oscillospiraceae*. Moreover, there is no reference genome, so it is difficult to study its function. However, the existing correlation analysis shows that short-chain fatty acid butyric acid may be produced by *Oscillospiraceae*, which is related to intestinal barrier function [28], suggesting that *Oscillospiraceae* plays a positive role in the maintenance of human health [29].

### 3.5. The Transplantation of Het Mouse Feces Reduces the Resistance of KO Mice to EHEC Infection

**Figure 5:**
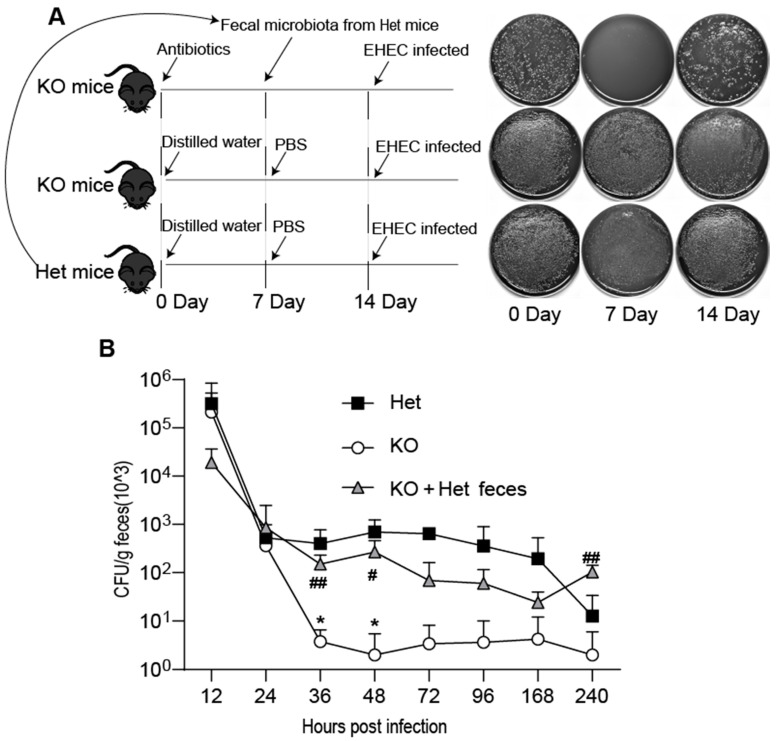
Verification of EHEC colonization ability after FMT. (**A**) Establishment of FMT. KO mice were used as recipients and Het mice were used as donors. Three groups of mice (*n* = 6) were processed following the schematic diagram (**left**), and feces were collected on 0 days, 7 days and 14 days, and cultured on Columbia plates (**right**). (**B**) EHEC infected these three groups at the same dose. The feces at different points were collected, made into suspensions, and applied to Sorbitol-MacConkey agar plates after serial dilution. *t*-test, * represents group KO compared to group Het, * *p* < 0.05; # epresents group “KO+Het feces” compared to group KO, ^#^ *p* < 0.05, ^##^ *p* < 0.01, error bars represent SEM. These experiments were repeated twice. KO, Knockout; Het, Heterozygote; EHEC, Enterohemorrhagic *Escherichia coli*; PBS, phosphate-buffered saline.

As *Akkermansia* and *Oscillospiraceae* bacteria are related to intestinal barrier function, in which *Akkermansia* can provide colonization resistance to pathogenic bacteria for the host. Both bacterial communities were increased simultaneously in KO mice gut, suggesting that this group of mice had a more agile resistance to pathogens. Next, we used mixed antibiotics to clean up the intestinal microbes of KO mice, and then transplant the feces of group Het to group KO (Figure 5A). In this way, a FMT model was constructed to explore the relationship between EHEC colonization and intestinal flora. We found that after EHEC infected the FMT model, the colonization ability was enhanced (Figure 5B). It shows that after the replacement of *Akkermansia* and *Oscillospiraceae* bacteria, the immunity of the intestinal mucosal barrier is reduced, and the invasiveness of pathogenic bacteria is enhanced. And at 10 days of infection, the FMT group showed a higher bacterial load than the Het group. The possible reason is that the lack of *Vsig4* was not conducive to the elimination of pathogen, and the abundance of probiotics (*Akkermansia* and *Oscillospiraceae*) were reduced, so it is more conducive to the colonization of EHEC.

The above results showed that molecule VSIG4 regulates intestinal anti-*Enterohemorrhagic Escherichia coli* immunity by altering gut flora diversity.

## 4. Discussion

Recently, it has been found that VSIG4 is not constitutively expressed in macrophages but depends on the external signal-induced expression [4] produced after birth. This study showed that VSIG4 was not expressed in neonatal mice but showed a high expression trend with age. Especially in the period before and after weaning, the expression of VSIG4 increased sharply. This period is when the early colonization of intestinal microorganisms plays a crucial role in the immune development of the body. And after continuous treatment with mixed antibiotics in adult mice, the expression of VSIG4 in macrophages was significantly decreased. This phenomenon suggested that microbial signals induce the expression of VSIG4 in tissues. However, in this study, the deletion of *Vsig4* can change the structure of intestinal flora in mice, indicating a two-way regulation between them.

Our study showed that deletion of *Vsig4* could increase intestinal microbiota diversity (Figure 2), which may be related to the its immunosuppressive function as a member of the B7 family. Immune tolerance mediated by B7 family molecule CTLA-4/PD-1 is necessary to protect and preserve probiotic microbial communities in the gastrointestinal tract [17]. And studies have shown that when immune checkpoints of the B7 family were suppressed; *Bifidobacterium pseudolongum*, *Lactobacillus johnsonii* and *Olsenella species* in the intestinal tract can significantly enhance anticancer immunity [18]. These studies show that there is a regulation and even antagonistic relationship between B7 family and the intestinal microbial community. Thus, the possible reasons for the increase of intestinal resistance to pathogenic bacteria in *Vsig4*^−/−^ mice are: (1) deletion *Vsig4* changed the intestinal flora structure. The increase of probiotic *Akkermansia* and *Oscillospiraceae* bacteria (Figure 3D) strengthened the intestinal mucosal barrier; (2) the loss of co-inhibitory molecule VSIG4 reduces the threshold of intestinal innate and cellular immune activation and increases the immune response, which is beneficial to resist the invasion of pathogenic bacteria. Notably, the researchers found that although the immune response was up-regulated in *Vsig4*^−/−^ mice, it did not translate into spontaneous inflammatory disease. They speculate that the reason may be the low level of inflammation under laboratory mouse keeping conditions or that there is a corresponding antagonistic mechanism [30]. Therefore, there was no difference in the level of serum KC in WT and KO mice. Only when infected with the pathogen EHEC, the difference in blood inflammation and colon pathology were reflected (Figure 1D,E). It is inferred that this difference is due to the degree of invasion of pathogens; that is, the root cause is the difference in intestinal resistance.

The FMT model established in this article mainly referred to the method of Wang et al. [31]. Although germ-free are the best model for FMT, the breeding is very demanding on the environment and requires specific qualifications. Therefore, in a relatively sterile breeding environment, many scientists choose antibiotic-treated mice to establish FMT model [31,32,33,34]. Using a cocktail of antibiotics can remove intestinal microbes very quickly, and it takes about five days for its own intestinal flora to recover (Supplementary Figure S5). This provides sufficient colonization time for the donor flora, which can be used as an effective acceptor.

At present, the traditional impression of VSIG4 molecule is that B7 family co-suppresses molecules and complement receptors. In this paper, it is proposed for the first time that VSIG4 is involved in the Regulation of mouse intestinal flora structure. The intestinal microflora is closely related to mucosal immunity and systemic immunity [35], suggesting that there may always be a “supporting role” in the multiple immune regulatory roles played by the VSIG4. However, the potential relationship and mechanism between them are still largely blank, worthy of our in-depth study in the future.

## Data Availability

The whole-genome sequences of the mice are deposited at GenBank under the BioProject accession number PRJNA734571.

## Supplementary Materials

The following are available online at https://www.mdpi.com/article/10.3390/microorganisms9081769/s1, Figure S1: Species accumulation box plot and Alpha diversity index between two groups, Figure S2: NMDS on OTU level, Figure S3: A. Community heatmap analysis on Phylum and Genus level, Figure S4: Ct curve, Figure S5: The recovery of the intestinal flora. Table S1: Primers and the data of PCR product.
