# Supplementary material for: B7 Family Molecule VSIG4 Regulates Intestinal Anti-Enterohemorrhagic Escherichia coli Immunity by Altering Gut Flora Diversity
Source: Microorganisms. 2021 Aug 19;9(8):1769. doi: 10.3390/microorganisms9081769 (PMC8398431; doi:10.3390/microorganisms9081769)
Supplement: Supplementary file 1 [file microorganisms-09-01769-s001.zip › microorganisms-1286196-supplementary/Supplementary File/Supplementary File.pdf]

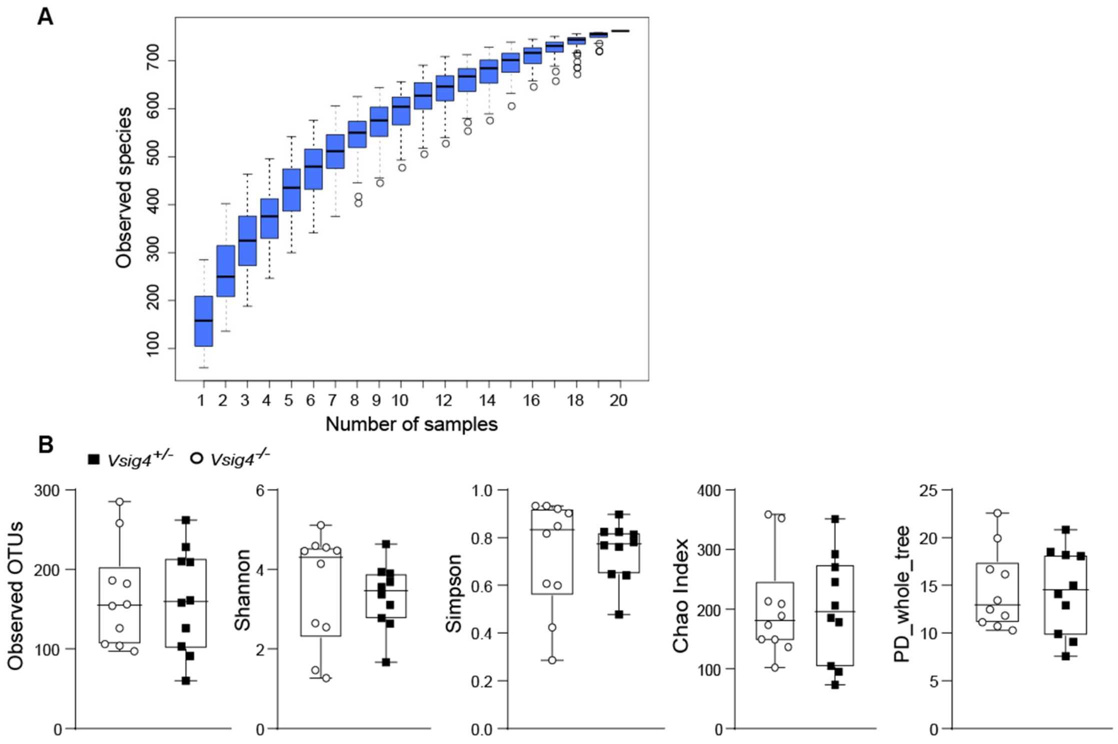

Supplementary Figure S1. A. Species accumulation box plot. The abscissa is the sample size; the ordinate is the number of OTUs after sampling. The results reflect the rate of emergence of new OTUs (new species) under continuous sampling.The species accumulation box chart can be used as a judgment of whether the sample size is sufficient. **B.** Alpha diversity index between two groups. Shannon and Simpson index reflects Community diversity; Chao index reflects Community richness; PD_whole_tree represents phylogenetic diversity index. Non-parametric Mann-Whitney U test.

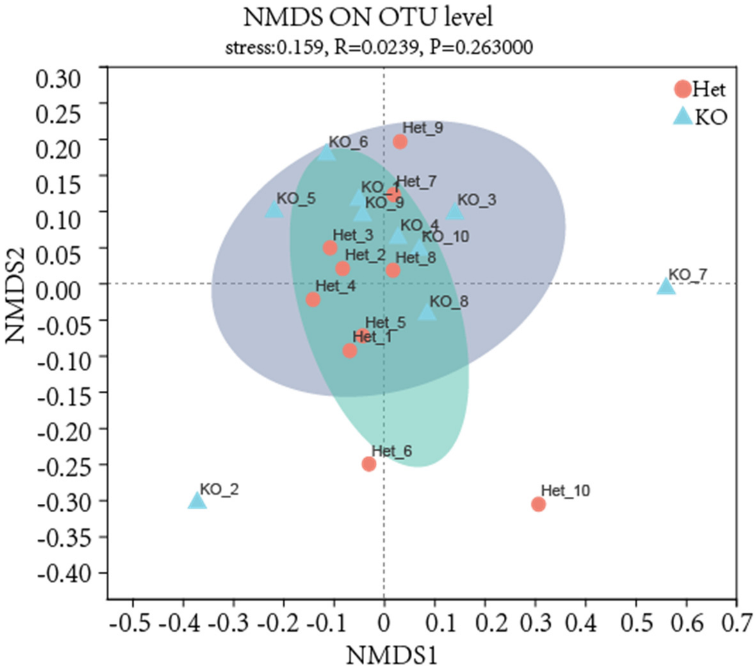

Supplementary Figure S2. NMDS on OTU level.

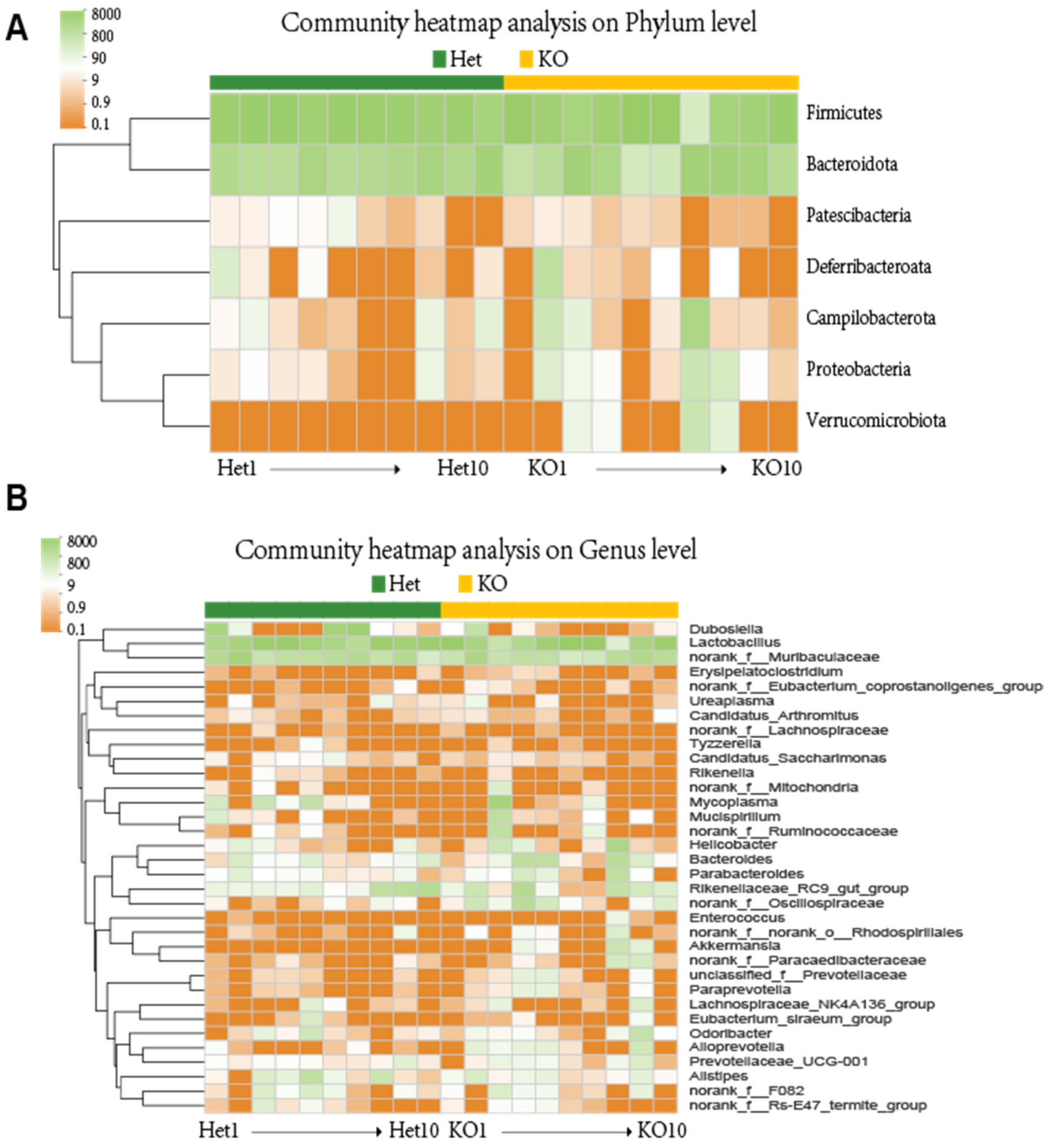

Supplementary Figure S3. Community heatmap analysis on Phylum level. **B.** Community heatmap analysis on Genus level.

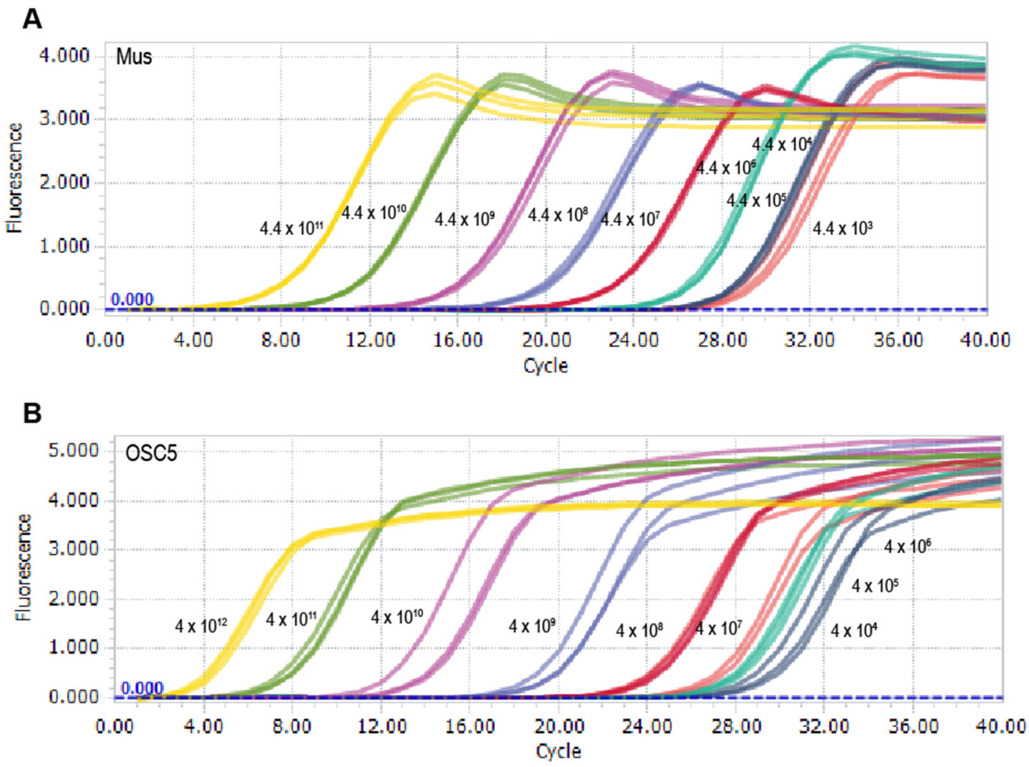

Supplementary Figure S4

Supplementary Table S1. Primers and the data of PCR product.

Names	Sequence(5'-3')	Specificity	PCR product		
			A_{260}/A_{280}	Concentration (g/mL)	Copies of target gene(Copies/ml)
Muc-F	cagcacgtgaaggtggggac	*Oscillospira*	2.0	1.5 x 10^{-7}	4.4x 10^{11}
Muc-R	ccttgcggttggcttcagat				
OSC5-F	agcggtggattatgtggttt	*Akkermansia*	2.5	1.0 x 10^{-6}	4x 10^{12}
OSC5-R	tttgtcaacggcagtctcg				

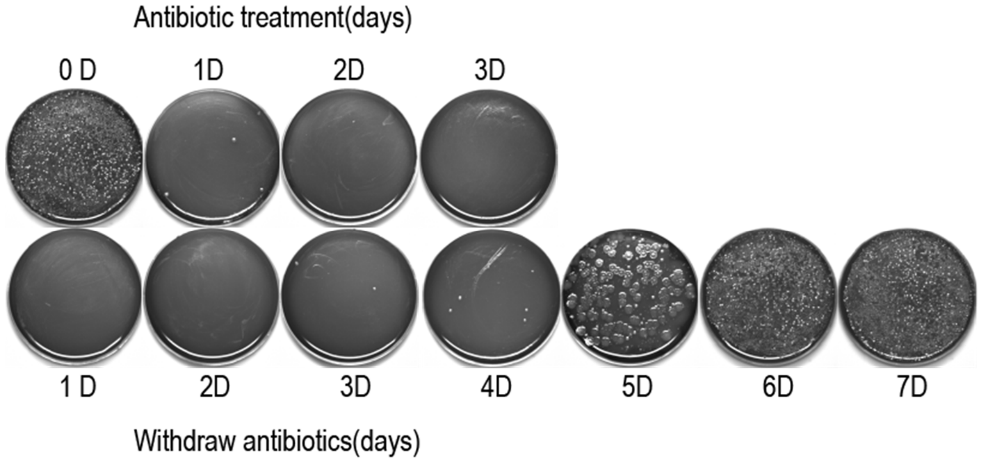

Supplementary Figure S5. The recovery of the intestinal flora.

Five KO mice were selected from 6-8 weeks old and treated them with cocktail antibiotics for three days. And randomly took the feces of three mice for seven consecutive days, prepared them as suspensions, and smeared them on Columbia blood plates for culture.
